# Risk of encephalitis and meningitis after COVID-19 vaccination in South Korea: a self-controlled case series analysis

**DOI:** 10.1186/s12916-024-03347-6

**Published:** 2024-03-14

**Authors:** Ju Hwan Kim, Dongwon Yoon, Hwa Yeon Ko, Kyungyeon Jung, Jun-Sang Sunwoo, Won Chul Shin, Jung-Ick Byun, Ju-Young Shin

**Affiliations:** 1 School of Pharmacy, Sungkyunkwan University, Suwon, South Korea; 2 Department of Biohealth Regulatory Science, Sungkyunkwan University, Suwon, South Korea; 3 Department of Neurology, Kangbuk Samsung Hospital, Seoul, South Korea; 4 Department of Neurology, Kyung Hee University Hospital at Gangdong, Kyung Hee University College of Medicine, Seoul, South Korea; 5 Department of Clinical Research Design & Evaluation, Samsung Advanced Institute for Health Sciences & Technology (SAIHST), Sungkyunkwan University, Seoul, South Korea

**Keywords:** COVID-19 vaccine, Encephalitis, Meningitis, Self-controlled case series

## Abstract

**Background:**

Several neurological manifestations shortly after a receipt of coronavirus infectious disease 2019 (COVID-19) vaccine have been described in the recent case reports. Among those, we sought to evaluate the risk of encephalitis and meningitis after COVID-19 vaccination in the entire South Korean population.

**Methods:**

We conducted self-controlled case series (SCCS) analysis using the COVID-19 immunization record data from the Korea Disease Control Agency between February 2021 and March 2022, linked with the National Health Insurance Database between January 2021 and October 2022. We retrieved all medical claims of adults aged 18 years or older who received at least one dose of COVID-19 vaccines (BNT162b2, mRNA-1273, ChAdOx1-S, or Ad26.COV2.S), and included only those who had a diagnosis record for encephalitis or meningitis within the 240-day post-vaccination period. With day 0 defined as the date of vaccination, risk window was defined as days 1–28 and the control window as the remainder period excluding the risk windows within the 240-day period. We used conditional Poisson regression to estimate the incidence rate ratios (IRR) with 95% confidence intervals (CI), stratified by dose and vaccine type.

**Results:**

From 129,956,027 COVID-19 vaccine doses administered to 44,564,345 individuals, there were 251 and 398 cases of encephalitis and meningitis during the risk window, corresponding to 1.9 and 3.1 cases per 1 million doses, respectively. Overall, there was an increased risk of encephalitis in the first 28 days of COVID-19 vaccination (IRR 1.26; 95% CI 1.08–1.47), which was only significant after a receipt of ChAdOx1-S (1.49; 1.03–2.15). For meningitis, no increased risk was observed after any dose of COVID-19 vaccine (IRR 1.03; 95% CI 0.91–1.16).

**Conclusions:**

Our findings suggest an overall increased risk of encephalitis after COVID-19 vaccination. However, the absolute risk was small and should not impede COVID-19 vaccine confidence. No significant association was found between the risk of meningitis and COVID-19 vaccination.

**Supplementary Information:**

The online version contains supplementary material available at 10.1186/s12916-024-03347-6.

## Background

Implementation of global mass vaccination campaign against coronavirus disease 2019 (COVID-19) has resulted in more than 5.5 billion people worldwide to receive a dose of COVID-19 vaccine [1]. The effectiveness of the approved COVID-19 vaccines in preventing death and post-sequalae from COVID-19 has been demonstrated extensively in the published studies [2, 3]. However, mass population exposure to COVID-19 vaccines has also led to the reporting of rare but serious adverse events that were not powered to be detected in a pre-authorization trial setting. This subsequently led regulatory agencies to launch COVID-19 vaccine safety surveillance strategies, one of which was to specify adverse events of special interest (AESIs) of COVID-19 vaccines and the evaluation for their causal association [4, 5].

Encephalitis and meningitis are inflammation of the brain parenchyma and meninges, respectively, and are reported as one of the AESIs of COVID-19 [6]. Several reports of post-vaccination encephalitis had been reported after influenza, hepatitis B or measles, mumps, and rubella vaccines [7, 8]. While definite biological plausibility remains unclear, a growing number of case reports suggest these events to be immune-mediated neurological acute sequelae after COVID-19 vaccination [9–11]. Compared with the other AESIs, there had been relatively few observational studies on these neurological events partly owing to very few cases recorded in the data. In a multi-national cohort study on the background incidence rates of the AESIs, the incidences of neurological events were extremely rare, occurring in < 1 case per 10,000 person-years [4]. A recent study in the United Kingdom (UK) population demonstrated a trend towards increased risk of composite outcome of encephalitis, meningitis, and myelitis after a first dose of ChAdOx1-S, yet the number of cases was too small to draw definitive conclusion on their causal association [12]. Previous reports evaluating public data reported a higher incidence of post-vaccination encephalitis after ChAdOx1 nCoV-19 vaccine but not after BNT162b2 [13]. However, it remains unclear whether the risk of encephalitis or meningitis persists beyond the first dose or differs across different COVID-19 vaccine platforms.

To address this research gap, we conducted a nationwide population-based study to evaluate the association between the two neurological events and COVID-19 vaccinations in the South Korea’s healthcare database using a self-controlled case series (SCCS) analysis. COVID-19 vaccination in South Korea began in March 2021, with more than 86% of the population completing the primary series with one of the following COVID-19 vaccines: BNT162b2 (Pfizer-BioNTech), mRNA-1273 (Moderna), ChAdOx1-S (Oxford-AstraZeneca), Ad26.COV2.S (Janssen/Johnson & Johnson) [14].

## Methods

### Data source

This study was a part of South Korea’s COVID-19 Vaccine Safety Research Committee (CoVaSC) for establishing evidence to guide safe use of COVID-19 vaccines. CoVaSC utilizes a large-linked database by linking the COVID-19 immunization registry data from the Korea Disease Control and Prevention Agency and the National Health Insurance System Database (NHID). The linkage between the two data sources is deterministic, using the 13-digit Resident Registration Number issued to all residents of South Korea. Briefly, COVID-19 immunization registry data was delivered to the secure data storage server of NHID, where medical claims of COVID-19 vaccine recipients were extracted. Patient consents were not required for the data linkage as pertinent data were anonymized.

Briefly, COVID-19 immunization registry data contains comprehensive information on the demographics (i.e., sex, age at immunization, and nationality), COVID-19 vaccine administration (i.e., vaccine type, dose number, administration route, and vaccination site), and COVID-19 infection (i.e., date of positive test). NHID contains all medical claims of the entire South Korean population, including demographics, income-based insurance premium tier as a proxy for socioeconomic status, diagnoses, procedures, in-patient medication orders, out-patient prescriptions, and date of death through linkage to the national statistics data. Medical diagnoses are recorded using the International Classification of Diseases, 10th revision (ICD-10). More details on the two data sources are described elsewhere [15, 16].

### Study population

From the large-linked database, we procured all claims data of 42 million individuals aged 18 years or older at their first COVID-19 vaccination between February 26, 2021, and October 31, 2022. Among them, we included those with a diagnosis record for encephalitis or meningitis after COVID-19 vaccination in the SCCS analysis.

Individuals who were vaccinated outside of South Korea, foreigners, or clinical trial enrollees were excluded as their healthcare utilization after COVID-19 vaccination may not be fully captured. Individuals with incomplete immunization record(s) such as missing vaccine type, dose number, etc. were also excluded to avoid any exposure misclassification. Individuals with a diagnosis record for encephalitis or meningitis in the 365 days prior to their first COVID-19 vaccine dose were excluded to capture only the incident outcomes. Lastly, those diagnosed with encephalitis or meningitis outside the hospital setting were excluded to improve the validity of the captured outcomes.

### Outcomes

The outcomes of interest were encephalitis and meningitis after a receipt of COVID-19 vaccine. These neurological events were captured using ICD-10 codes and restricted to diagnoses made during hospitalization. To further improve the outcome validity, we required patients to have a procedural code for cerebrospinal fluid (CSF) test with the diagnosis record. This operational definition was adopted from previous literature and Brighton Collaboration case definitions for acute encephalitis and meningitis published by the Safety Platform for Emergency vACcines (SPEAC) [12, 17, 18], which also has been reviewed by the CoVaSC clinical research committee (Additional file 1: Table S1).

### Self-controlled case series analysis

We used SCCS analysis to determine whether there is a transient increased risk of study outcome after COVID-19 vaccination. This analysis uses data from individuals who had experienced both exposure and outcome of interest in a pre-specified observation period. Comparison is made within the individual by comparing outcome incidence rates in a risk window on or after the date of exposure with a control window assumed to be unrelated to exposure. As the individuals are acting as their own control, this design inherently controls for time-fixed confounders such as demographics and pre-existing chronic medical conditions. Further details on SCCS design for vaccine safety research are described in depth elsewhere [19, 20].

The observation period was defined as a 240-day period from the date of the first COVID-19 vaccination. The risk window was defined as 1–28 days after each dose, and the control window as the remainder days within the observation period; the 28-day window was based on a systemic review of case reports that described an onset of encephalitis ranging from 1 to 30 days after COVID-19 vaccination [21]. To meet the assumption of SCCS analysis on independency between outcome and subsequent exposure, only the first outcome was included.

### Statistical analysis

Descriptive analysis was conducted to summarize the characteristics of encephalitis and meningitis cases, stratified by outcome occurrence in the risk or control periods, using mean with standard deviation (SD) for continuous variables and frequencies with percentages for categorical variables. Demographics including age, sex, health insurance type, and residences were ascertained on the date of the first COVID-19 vaccination. Medical claims 365 days prior to the vaccination were analyzed to measure the Charlson comorbidity index (CCI) score and comorbidities including myocardial infarction, congestive heart failure, peripheral vascular disease, cerebrovascular disease, dementia, chronic pulmonary disease, rheumatic disease, peptic ulcer disease, hepatic disease, diabetes mellitus, renal disease, cancer and Human immunodeficiency virus infection.

For the SCCS analysis, the number of events and time under exposure were measured to estimate the incidence rate per person-year in the risk and control windows. The conditional Poisson regression model was used to estimate the incidence rate ratios (IRR) and 95% confidence intervals (CI), comparing the rate in the risk window with the control window, for each outcome of interest. The IRRs were presented overall, and stratified by scheduled dose (i.e., first, second, and third doses) and vaccine type, and post-hoc subgroup analyses were conducted according to age group (10-year bands), sex, and comorbidities.

We conducted sensitivity analyses to test the robustness of findings against various assumptions made in this study. First, to test the impact of different risk window lengths on the study outcomes, we repeated the analyses using the shorter and longer risk windows of 14 days and 42 days, respectively. Second, to test the robustness of case definitions for capturing the study outcomes, analyses were conducted by: (1) restricting encephalitis and meningitis cases with a diagnosis record at the primary position; (2) restricting encephalitis cases with a prescription record for antivirals, intravenous immunoglobulin or systemic corticosteroids on the date of diagnosis; (3) including encephalitis and meningitis cases with a diagnosis made not only in-hospital but also at emergency department; (4) excluding encephalitis and meningitis cases identified using the diagnosis codes for bacterial or viral encephalitis/meningitis (ICD-10: A87, G04.2). Third, to account for the positive association between encephalitis/meningitis and COVID-19 infection reported previously [6, 12], analyses were conducted by: (1) excluding cases with positive COVID-19 test 90 days prior to vaccination; (2) excluding cases with positive COVID-19 test prior to diagnosis of encephalitis or meningitis. Lastly, to meet the assumption of SCCS analysis that an event should not censor the observation period, we repeated the analysis by excluding cases who died during the 240-day observation period.

The analyses were conducted separately for encephalitis and meningitis by constructing two separate case groups for SCCS analysis. All analyses were conducted using SAS version 9.4 (SAS Institute Inc., Cary, NC, USA), and a 2-sided α of less than 0.05 was considered statistically significant.

## Results

**Fig. 1:**
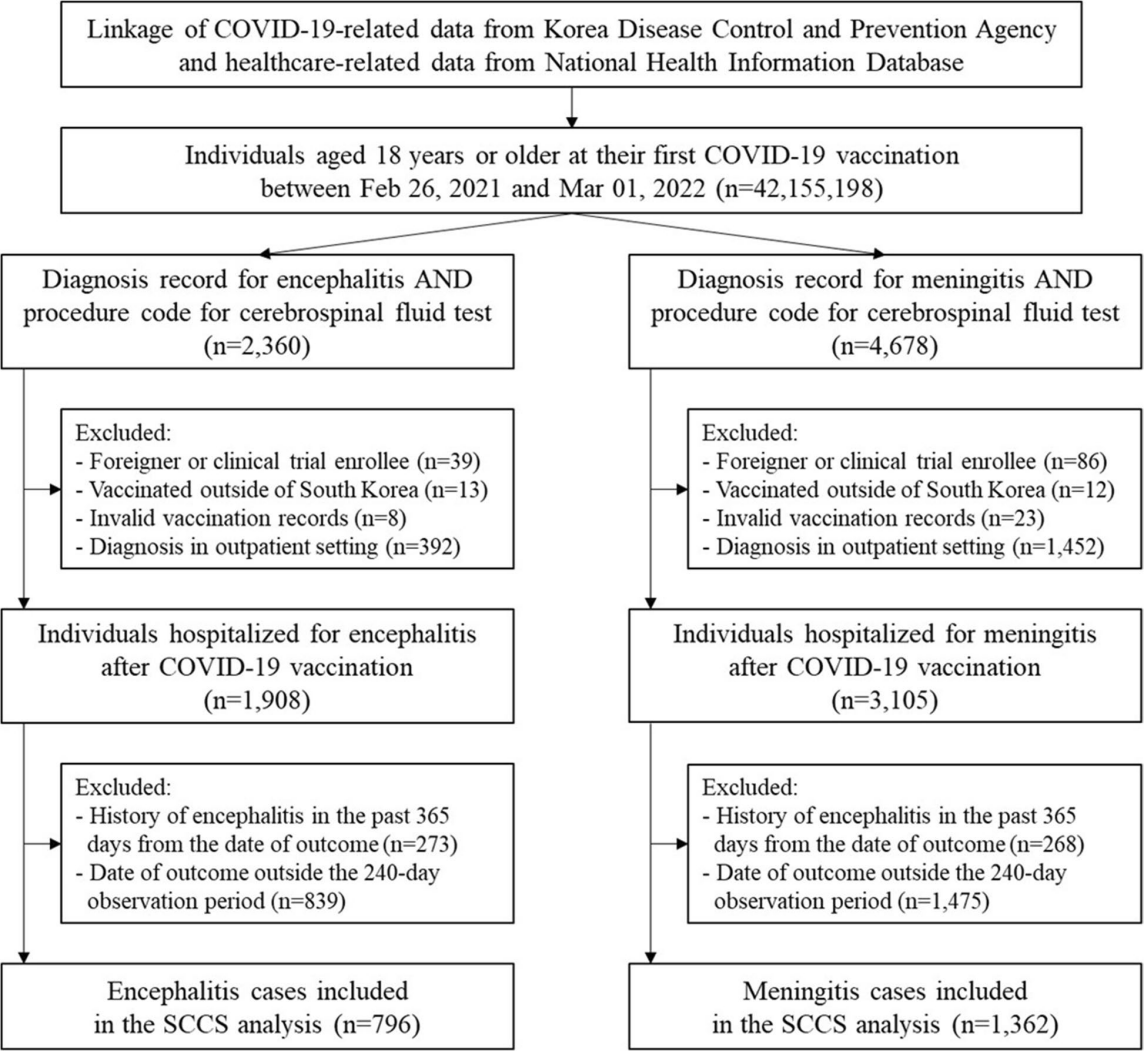
Study flow chart. Abbreviations: COVID-19, coronavirus infectious disease 2019; SCCS, self-controlled case series

From 129,956,027 COVID-19 vaccine doses administered to 44,564,345 individuals, there were 796 encephalitis and 1,362 meningitis cases included in the SCCS analysis (Fig. 1).

### Encephalitis

Of the encephalitis cases, there were 251 cases identified in the risk window, corresponding to 1.9 cases per 1 million doses. Their mean age (SD) at first COVID-19 vaccination was 56.9 years (18.0) and 57.8% were men (Table 1).

**Table 1:** Baseline characteristics of encephalitis and meningitis cases after COVID-19 vaccination, stratified by exposure windows

Characteristic	Encephalitis		*P*-value	Meningitis		*P*-value
	**Risk window** **(Days 1–28)**	**Control window**^**a**^ **(All other periods)**		**Risk window** **(Days 1–28)**	**Control window**^**a**^ **(All other periods)**	
	***N* (%)**	***N* (%)**		***N* (%)**	***N* (%)**	
**Total**	**251 (100.0)**	**545 (100.0)**		**398 (100.0)**	**964 (100.0)**	
**Age, years (mean, SD)**	56.9 (18.0)	56.3 (18.8)	0.722	45.1 (18.8)	44.7 (18.7)	0.671
18–29	33 (13.1)	72 (13.2)	0.342	109 (27.4)	270 (28)	0.747
30–39	12 (4.8)	40 (7.3)		75 (18.8)	181 (18.8)	
40–49	30 (12.0)	71 (13)		57 (14.3)	139 (14.4)	
50–59	46 (18.3)	92 (16.9)		45 (11.3)	128 (13.3)	
60–69	67 (26.7)	109 (20)		64 (16.1)	122 (12.7)	
70–79	43 (17.1)	110 (20.2)		34 (8.5)	87 (9)	
80 +	20 (8.0)	51 (9.4)		14 (3.5)	37 (3.8)	
**Sex**			0.270			0.518
Male	145 (57.8)	292 (53.6)		200 (50.3)	503 (52.2)	
Female	106 (42.2)	253 (46.4)		198 (49.7)	461 (47.8)	
**Health insurance type**			0.782			0.080
NHI	235 (93.6)	513 (94.1)		381 (95.7)	940 (97.5)	
Medical aid	16 (6.4)	32 (5.9)		17 (4.3)	24 (2.5)	
**Region of residence**			0.314			0.268
Metropolitan	182 (72.5)	376 (69)		287 (72.1)	666 (69.1)	
Rural	69 (27.5)	169 (31)		111 (27.9)	298 (30.9)	
**Comorbidities**						
CCI (mean, SD)	2.0 (2.6)	1.9 (2.3)	0.771	1.3 (2.2)	1.2 (2)	0.345
CCI < 5	219 (87.3)	479 (87.9)	0.799	357 (89.7)	899 (93.3)	0.026
CCI ≥ 5	32 (12.7)	66 (12.1)		41 (10.3)	65 (6.7)	
Myocardial infarction	5 (2.0)	4 (0.7)	0.119	4 (1)	7 (0.7)	0.601
Congestive heart failure	17 (6.8)	36 (6.6)	0.930	20 (5)	22 (2.3)	0.008
Peripheral vascular disease	36 (14.3)	83 (15.2)	0.744	31 (7.8)	79 (8.2)	0.803
Cerebrovascular disease	36 (14.3)	64 (11.7)	0.304	26 (6.5)	58 (6)	0.719
Dementia	22 (8.8)	51 (9.4)	0.788	19 (4.8)	41 (4.3)	0.670
Chronic pulmonary disease	50 (19.9)	112 (20.6)	0.837	64 (16.1)	150 (15.6)	0.810
Rheumatic disease	9 (3.6)	32 (5.9)	0.175	18 (4.5)	26 (2.7)	0.083
Peptic ulcer disease	40 (15.9)	101 (18.5)	0.373	70 (17.6)	138 (14.3)	0.127
Hepatic disease	63 (25.1)	129 (23.7)	0.661	73 (18.3)	188 (19.5)	0.621
Diabetes mellitus	57 (22.7)	141 (25.9)	0.338	59 (14.8)	146 (15.1)	0.880
Renal disease	8 (3.2)	30 (5.5)	0.154	9 (2.3)	27 (2.8)	0.572
Cancer	25 (10.0)	35 (6.4)	0.079	26 (6.5)	43 (4.5)	0.113
HIV infection	1 (0.4)	0 (-)	0.140	1 (0.3)	3 (0.3)	0.853

*Abbreviations*: *COVID-19* Coronavirus infectious disease 2019, *SD* Standard deviation, *NHI* National Health Insurance, *CCI* Charlson comorbidity index, *HIV* Human immunodeficiency virus

^a^Control window was defined as 29 days to 240 days after the exposure date (i.e., COVID-19 vaccination date), censored on the date of death

Overall, there was an increased risk of encephalitis in the first 28 days after any doses of COVID-19 vaccine (IRR 1.26; 95% CI 1.08–1.47). When stratified by scheduled dose, there was a trend towards increased risk for first dose (IRR 1.19; 95% CI 0.97–1.47), whereas no risk for the second (0.96; 0.76–1.20) and third doses (0.92; 0.67–1.26). In the stratified analysis by vaccine type, significant risk was present only after a receipt of ChAdOx1-S (IRR 1.49; 95% CI 1.03–2.15) (Table 2).

**Table 2:** Risk of encephalitis after COVID-19 vaccination, overall and stratified by scheduled dose and vaccine type

	**No. event**		**Person-years**		**Incidence rate**^**a**^		**IRR (95% CI)**
	**Risk**	**Control**	**Risk**	**Control**	**Risk**	**Control**	
**Encephalitis**							
**Overall**	251	545	134.8	369.3	1.86	1.48	1.26 (1.08–1.47)
**Scheduled dose**							
1st dose	106	545	60.2	369.3	1.76	1.48	1.19 (0.97–1.47)
2nd dose	87	438	53.2	256.5	1.63	1.71	0.96 (0.76–1.20)
3rd dose	58	83	21.3	28.2	2.72	2.94	0.92 (0.67–1.26)
**Vaccine type**							
BNT162b2	123	279	69.2	188.0	1.78	1.48	1.20 (0.96–1.49)
mRNA-1273	21	62	13.6	36.5	1.54	1.70	0.91 (0.54–1.52)
ChAdOx1-S	44	110	19.7	73.1	2.23	1.50	1.49 (1.03–2.15)
Ad26.COV2.S	0	7	0.5	3.4	-	2.07	-

*Abbreviations*: *COVID-19* Coronavirus infectious disease 2019, *IRR* Incidence rate ratio, *CI* Confidence interval

^a^Per person-year

While the risks were generally non-significant in the majority of subgroups owing to few cases in each stratum, the point estimates were largely consistent with the main findings. Notably, the risk was significant among those aged between 60 and 69 years (IRR 1.64; 95% CI 1.21–2.24), with underlying cerebrovascular disease (1.57; 1.05–2.37), and cancer (1.94; 1.16–3.25) (Additional file 1: Table S2). The results of sensitivity analyses remained largely consistent with the main findings; the risk remained significant using the shorter and longer risk windows of 14 days (IRR 1.22; 95% CI 1.01–1.46) and 42 days (1.30; 1.13–1.50), respectively (Additional file 1: Table S3–S5).

### Meningitis

For meningitis, there were 398 cases in the risk window, corresponding to 3.1 cases per 1 million doses. Meningitis cases were relatively young, with a mean age (SD) of 45.1 years and 50.3% were men (Table 1).

There was no increased risk of meningitis in the first 28 days after any doses of COVID-19 vaccine (IRR 1.03; 95% CI 0.91–1.16), and the findings remained consistent when stratified by scheduled dose (1.10; 0.94–1.29 for first dose; 0.91; 0.76–1.09 for second dose; and 0.76; 0.57–1.00 for third dose). In the stratified analysis by vaccine type, the risk tended to associate with non-mRNA-based vaccines, with significant risk noted after a receipt of Ad26.COV2.S (IRR 4.22; 95% CI 1.22–14.59) and a trend towards increased risk for ChAdOx1-S (1.45; 0.99–2.11) (Table 3).

**Table 3:** Risk of meningitis after COVID-19 vaccination, overall and stratified by scheduled dose and vaccine type

	**No. event**		**Person-years**		**Incidence rate**^**a**^		**IRR (95% CI)**
	**Risk**	**Control**	**Risk**	**Control**	**Risk**	**Control**	
**Meningitis**							
**Overall**	398	964	252.8	628.9	1.57	1.53	1.03 (0.91–1.16)
**Scheduled dose**							
1st dose	178	964	105.4	628.9	1.69	1.53	1.10 (0.94–1.29)
2nd dose	147	784	98.3	478.1	1.50	1.64	0.91 (0.76–1.09)
3rd dose	73	134	49.2	68.6	1.49	1.95	0.76 (0.57–1.00)
**Vaccine type**							
BNT162b2	213	509	137.1	326.9	1.55	1.56	1.00 (0.85–1.17)
mRNA-1273	59	156	41.9	104.4	1.41	1.49	0.94 (0.69–1.29)
ChAdOx1-S	41	106	19.3	72.3	2.12	1.47	1.45 (0.99–2.11)
Ad26.COV2.S	4	7	0.8	6.2	4.74	1.12	4.22 (1.22–14.59)

*Abbreviations*: *COVID-19* Coronavirus infectious disease 2019, *IRR* Incidence rate ratio, *CI* Confidence interval

^a^Per person-year

In the subgroup analysis, an increased risk of meningitis was observed among those with CCI score of 5 or higher (IRR 1.54; 95% CI 1.04–2.29) or congestive heart failure (2.18; 1.20–3.95), whereas null association was found across all other selected subgroups (Additional file 1: Table S6). The results of sensitivity analyses remained largely consistent with the main findings (Additional file 1: Table S7–S9).

## Discussion

In this nationwide population-based study using a large-linked database between the national COVID-19 immunization registry and administrative claims data, we found a positive association for encephalitis in the COVID-19-vaccinated adults between 2021 and 2022. We observed a transient risk of post-vaccination encephalitis, which was mainly driven by the events recorded in the 28 days after the first dose and ChAdOx1-S. While no significant risk of meningitis was found in the combined analysis of all COVID-19 vaccine doses, there was a heterogeneity in the risk across the vaccine type, with the significant risk observed after a receipt of Ad26.COV2.S.

Our findings on the risk of encephalitis after COVID-19 vaccination complement the previously reported trends toward increased risk in the population-based studies. In a study of 4.3 million adults vaccinated with ChAdOx1-S recorded in the UK primary care records data between December 2020 and May 2021, standardized IRR of post-vaccination encephalomyelitis, compared with pre-pandemic era (2017–2019), was 1.45 (95% CI 0.80–2.62) [4]. Another study in the UK identified 282 cases composite of encephalitis, meningitis and myelitis in the 28-day period after COVID-19 vaccination, corresponding to an incidence rate of 8.7 cases per 1 million doses; risk of the composite outcome was assessed using SCCS analysis, revealing a trend towards increased risk 8–14 days after one dose of ChAdOx1-s (IRR 1.32; 95% CI 0.99–1.76) [12]. In view of these findings, we procured the latest available data to include more COVID-19 vaccine episodes in the study analysis, addressing the limited statistical power noted from the past studies. Our findings confirm the trend in risk reported by previous studies and further suggest that the risk may be attributed to post-vaccination encephalitis.

Our finding on the null association between meningitis and COVID-19 vaccination was certainly unexpected, especially given that encephalitis and meningitis share similarities in clinical presentation and etiology [22]. Our rationale for assessing these two potential neurological complications of COVID-19 vaccination separately was twofold: there had been several case reports describing them independently, and no large population-based studies to date have assessed them separately. In these contexts, our finding reassures the COVID-19 vaccine safety against post-vaccination meningitis. Nonetheless, there remains a need for additional studies to ascertain the association observed in this study. Meanwhile, clinicians should be advised of these COVID-19 vaccine-related neurological complications.

There was a heterogeneity in the risk of encephalitis and meningitis according to the type of COVID-19 vaccines. Existing data have indicated better safety profiles for the mRNA-based vs. non-mRNA-based vaccines [23]. In a study comparing cytokine concentrations pre- and post-COVID-19 vaccination among individuals without a history of COVID-19 positive test, inflammation markers (i.e., c-reactive protein, tumor necrosis factor-α, interleukins-1β, 6, 8, and 10) were elevated 8–16 days post-vaccination, compared with pre-vaccination period, and larger increase noted for adenovirus-based compared to mRNA-based vaccines [24]. In a cohort study that examined the association between Guillain-Barré syndrome (GBS), another major neurological AESI, and COVID-19 vaccination in the US Vaccine Safety Datalink, the risk of GBS after Ad26.COV2.S was 20-fold higher, compared with mRNA-based vaccines (i.e., BNT162b2 and mRNA-1273) [25]. Our findings on the association of non-mRNA-based vaccines with encephalitis and meningitis are generally in line with the existing data that showed an elevated risk of immune-mediated neurological events with adenovirus-based vaccines [4, 12]. However, the observed trend towards increased risk of encephalitis after BNT162b2 may also suggest that the risk of immune-mediated neurological events may be present regardless of the types of COVID-19 vaccine. Moreover, it remains unclear whether the risk of post-vaccination encephalitis or meningitis is specific to COVID-19 vaccines as these events had previously been linked to routinely administered vaccines [8, 26, 27].

Emerging data suggests that encephalitis and meningitis after COVID-19 vaccination are non-infectious immune-mediated conditions, with proposed mechanisms including direct triggering of inflammatory cytokines by the vaccine and molecular mimicry between SARS-CoV-2 spike protein and myelin basic protein [28, 29]. These immune-mediated mechanisms are supported by favorable outcomes after immunosuppressive therapy observed among patients with post-vaccination encephalitis or meningitis. In a systematic review of the case reports of 65 patients treated for COVID-19 vaccine-induced encephalitis, 49.2% had pleocytosis in their CSF, 86.2% were treated with systemic steroids or other immunosuppressants, and 63.1% achieved a full recovery [21]. In another case report, a young male patient in South Korea admitted for meningitis after receiving BNT162b2 had positive SARS-CoV-2 immunoglobulin G (IgG) in CSF, and was treated successfully with intravenous methylprednisolone [11]. Similarly, an encephalitis case following mRNA-1273 was unresponsive to antiviral treatment initially, but achieved full recovery after switching to pulse corticosteroid therapy [30]. Cases of post-vaccination encephalitis and meningitis following receipt of non-mRNA-based vaccines also have been described, in which all patients were treated successfully with immunosuppressive therapy after ruling out pathogen-induced encephalitis [13].

In the subgroup analyses, we observed patients with pre-existing comorbidities including cerebrovascular disease, congestive heart failure, and malignancy were at increased risk of post-vaccination encephalitis or meningitis. Assuming that all identified cases were triggered by systemic inflammatory response against COVID-19 vaccine, these comorbidities might have predisposed patients at a greater risk of post-vaccination encephalitis or meningitis. For instance, blood–brain barrier disruption from prolonged brain ischemia can lead patients with cerebrovascular diseases susceptible to neuroinflammation [31]. Autoimmune encephalitis manifesting from paraneoplastic syndromes is not uncommon in patients with solid tumors, although the impact of COVID-19 vaccination in this context remains to be explored [32]. It should also be noted that the observed risk in these patient groups requires careful interpretation as we did not account for disease severity and duration across the subgroups. Moreover, benefit from COVID-19 vaccination is likely to outweigh the potential risk of encephalitis and meningitis in these patient groups, especially given the current data showing an increased risk of poor prognosis from COVID-19 in patients with pre-existing comorbidities [33].

In this study, we presented real-world evidence on the safety of COVID-19 vaccinations through an assessment of the risk of the post-vaccination encephalitis or meningitis using the large-linked database representative of entire South Korean adults who received at least one dose of COVID-19 vaccines. The large sample size enabled us to estimate precise risk estimates for each outcome according to vaccine type, which could not be assessed in the clinical trials or observational studies with limited statistical power. The use of SCCS analysis allowed us to avoid bias arising from comparing COVID-19 vaccinated vs. unvaccinated individuals, and the within individual comparison removed any potential for confounding by time-invariant confounders.

There are several limitations to be considered when interpretating our study’s findings. First, there is a potential for misclassification or inaccuracy of the captured outcomes as we relied on the ICD-10 codes for case definition, and this may either over- or under-estimate the true incidences of post-vaccination encephalitis and meningitis. To address this, we have adopted definitions from the previous literature and case definition published by the Brighton Collaboration, thereby restricting our cases to those diagnosed in hospital settings and received CSF tests. Nonetheless, it should be noted that the exact etiology or subtypes of encephalitis and meningitis could not be ascertained due to the lack of imaging or CSF results in the database. Specifically, we could not differentiate between autoimmune and infectious encephalitis or meningitis, and thus, it is certainly possible that pathogens, not COVID-19 vaccine, may be responsible for some of the cases identified after COVID-19 vaccination. Second, there may be a delay between the actual disease onset and diagnosis recorded in the database, and this may have impacted our study findings if the onset and diagnosis did not occur in the risk window. However, the results from sensitivity analysis by varying the length of risk windows were comparable to that of the main analysis. Third, the impact of concurrent COVID-19 infection on our findings cannot be fully ruled out. COVID-19 itself has been shown to be associated with neurological complications [34]. We attempted to address this issue by excluding individuals with positive COVID-19 tests during the study period, but residual confounding may remain if an infected individual did not receive a COVID-19 test, and was later hospitalized for neurological complications after a seroconversion. Lastly, the legacy effect of COVID-19 vaccines on the risk of encephalitis or meningitis may have led to outcome misclassification. For instance, if the risk was to persist beyond the 28-day risk window after each COVID-19 vaccine dose, it may be certainly possible that encephalitis or meningitis elicited by the first dose may have been erroneously attributed to the subsequent doses. This could impact our findings according to the vaccine type, especially in cases involving mixed types of COVID-19 vaccines. In South Korea, ChAdOx1-S was used dominantly in the early 2021, which was shortly replaced with mRNA-based vaccines after a risk of thromboembolic events was unveiled with ChAdOx1-S [35]. In this context, along with the observed risk after a receipt of ChAdOx1-S in this study, encephalitis or meningitis cases were identified shortly after receiving mRNA-based vaccines as the second dose may be due to legacy effect carried over from the first dose of ChAdOx1-s. While the association between heterologous COVID-19 vaccination and risk of neurological complications was outside the scope of this study, future studies on vaccine safety should account for the legacy effect of COVID-19 vaccination.

## Conclusions

This large population-based study assessed the risk of two rare neurological complications after COVID-19 vaccination. Our findings underscore a transient risk of post-vaccination encephalitis and meningitis, which were likely to be attributed to the first scheduled dose and non-mNRA platform COVID-19 vaccines. However, it is important to note that the absolute risk was small and should not impede COVID-19 vaccine confidence. Rather, the finding is to inform clinical practice to enable prompt diagnosis and treatment for patients presenting with neurological signs after COVID-19 vaccination. Moreover, further studies are needed to confirm the differential magnitude of the risk estimates for encephalitis and meningitis observed in this study as these two neurological events share similar etiology.

### Supplementary Information

**Additional file 1: Table S1.** Diagnostic and procedure codes for encephalitis and meningitis in the national health insurance system database. **Table S2.** Subgroup analysis on the risk of encephalitis after COVID-19 vaccination according to the selected characteristics. **Table S3.** Sensitivity analysis on the risk of encephalitis after COVID-19 vaccination by varying the risk window lengths. **Table S4.** Sensitivity analysis on the risk of encephalitis after COVID-19 vaccination by varying the case definition.**Table S5.** Sensitivity analysis on the risk of encephalitis after COVID-19 vaccination in the modified study population. **Table S6.** Subgroup analysis on the risk of meningitis after COVID-19 vaccination according to the selected characteristics. **Table S7.**Sensitivity analysis on the risk of meningitis after COVID-19 vaccination by varying the risk window lengths. **Table S8.** Sensitivity analysis on the risk of meningitis after COVID-19 vaccination by varying the case definition. **Table S9.** Sensitivity analysis on the risk of meningitis after COVID-19 vaccination in the modified study population.

## Data Availability

The data from this study are available from the Korea Disease Control and Prevention Agency and National Health Insurance Service. While legal data-sharing agreements prohibit data from being made publicly available, access may be granted upon reasonable request with specific data needs, analysis plans, and dissemination plans submitted to the Korea Disease Control and Prevention Agency and National Health Insurance Service.

## Abbreviations

AESI: Adverse event of special interest
CCI: Charlson comorbidity index
CI: Confidence interval
CoVaSC: COVID-19 Vaccine Safety Research Committee (CoVaSC)
COVID-19: Coronavirus infectious disease 2019
CSF: Cerebrospinal fluid
GBS: Guillain-Barré syndrome
ICD-10: International Classification of Diseases, 10th revision
IgG: Immunoglobulin G
IRR: Incidence rate ratio
NHID: National Health Insurance System Database
SARS-CoV-2: Severe acute respiratory syndrome coronavirus 2
SCCS: Self-controlled case series
SD: Standard deviation
SPEAC: Safety Platform for Emergency vACcines
UK: United Kingdom
