# Supplementary material for: Risk of encephalitis and meningitis after COVID-19 vaccination in South Korea: a self-controlled case series analysis
Source: BMC Med. 2024 Mar 14;22:123. doi: 10.1186/s12916-024-03347-6 (PMC10941581; doi:10.1186/s12916-024-03347-6)
Supplement: Supplementary file 1 — Additional file 1: Table S1. Diagnostic and procedure codes for encephalitis and meningitis in the national health insurance system database. Table S2. Subgroup analysis on the risk of encephalitis after COVID-19 vaccination according to the selected characteristics. Table S3. Sensitivity analysis on the risk of encephalitis after COVID-19 vaccination by varying the risk window lengths. Table S4. Sensitivity analysis on the risk of encephalitis after COVID-19 vaccination by varying the case definition.Table S5. Sensitivity analysis on the risk of encephalitis after COVID-19 vaccination in the modified study population. Table S6. Subgroup analysis on the risk of meningitis after COVID-19 vaccination according to the selected characteristics. Table S7.Sensitivity analysis on the risk of meningitis after COVID-19 vaccination by varying the risk window lengths. Table S8. Sensitivity analysis on the risk of meningitis after COVID-19 vaccination by varying the case definition. Table S9. Sensitivity analysis on the risk of meningitis after COVID-19 vaccination in the modified study population. [file 12916_2024_3347_MOESM1_ESM.docx]

**Supplementary Table 1.** Diagnostic and procedure codes for encephalitis and meningitis in the national health insurance system database

| **ICD-10 code** | **Description** |
| --- | --- |
| G04 | Encephalitis, myelitis and encephalomyelitis |
| G05 | Encephalitis, myelitis and encephalomyelitis in disease classified elsewhere |
| A87 | Viral meningitis |
| G03 | Meningitis due to other and unspecified causes |
| G04.2 | Bacterial meningoencephalitis and meningomyelitis, not elsewhere specified |
| **Procedure code** |  |
| C8000 | Cerebrospinal fluid test |

Abbreviations: ICD-10, International Classification of Diseases.

**Supplementary Table 2.** Subgroup analysis on the risk of encephalitis after COVID-19 vaccination according to the selected characteristics

|  | **No. Event** | | **Person-Years** | | **IR** | | **IRR (95% CI)** |
| --- | --- | --- | --- | --- | --- | --- | --- |
|  | **Risk** | **Control** | **Risk** | **Control** | **Risk** | **Control** |  |
| **Encephalitis** |  |  |  |  |  |  |  |
| **Age group** |  |  |  |  |  |  |  |
| 18-29 | 33 | 72 | 17.9 | 51.4 | 1.84 | 1.40 | 1.31 (0.86-2.00) |
| 30-39 | 12 | 40 | 8.9 | 27.2 | 1.35 | 1.47 | 0.92 (0.47-1.81) |
| 40-49 | 30 | 71 | 17.5 | 47.1 | 1.71 | 1.51 | 1.14 (0.73-1.77) |
| 50-59 | 46 | 92 | 24.6 | 62.3 | 1.87 | 1.48 | 1.27 (0.87-1.83) |
| 60-69 | 67 | 109 | 30.7 | 82.0 | 2.19 | 1.33 | 1.64 (1.21-2.24) |
| 70-79 | 43 | 110 | 24.9 | 68.9 | 1.73 | 1.60 | 1.08 (0.76-1.54) |
| 80+ | 20 | 51 | 10.3 | 30.4 | 1.94 | 1.68 | 1.15 (0.69-1.94) |
| **Sex** |  |  |  |  |  |  |  |
| Male | 145 | 292 | 74.1 | 198.4 | 1.96 | 1.47 | 1.33 (1.09-1.63) |
| Female | 106 | 253 | 60.6 | 170.9 | 1.75 | 1.48 | 1.18 (0.93-1.49) |
| **Comorbidities** |  |  |  |  |  |  |  |
| CCI <5 | 219 | 479 | 118.7 | 327.6 | 1.85 | 1.46 | 1.26 (1.07-1.49) |
| CCI ≥5 | 32 | 66 | 16.1 | 41.7 | 1.99 | 1.58 | 1.26 (0.82-1.92) |
| Myocardial infarction | 5 | 4 | 1.5 | 3.7 | 3.30 | 1.09 | 3.02 (0.87-10.43) |
| Congestive heart failure | 17 | 36 | 8.7 | 22.9 | 1.96 | 1.57 | 1.25 (0.70-2.24) |
| Peripheral vascular disease | 36 | 83 | 19.4 | 52.9 | 1.86 | 1.57 | 1.18 (0.79-1.78) |
| Cerebrovascular disease | 36 | 64 | 16.0 | 44.7 | 2.25 | 1.43 | 1.57 (1.05-2.37) |
| Dementia | 22 | 51 | 11.7 | 32.3 | 1.87 | 1.58 | 1.19 (0.72-1.96) |
| Chronic pulmonary disease | 50 | 112 | 26.3 | 71.6 | 1.90 | 1.56 | 1.21 (0.86-1.71) |
| Rheumatic disease | 9 | 32 | 7.2 | 18.9 | 1.26 | 1.69 | 0.74 (0.36-1.54) |
| Peptic ulcer disease | 40 | 101 | 23.9 | 64.2 | 1.67 | 1.57 | 1.06 (0.73-1.55) |
| Hepatic disease | 63 | 129 | 33.0 | 84.2 | 1.91 | 1.53 | 1.25 (0.92-1.70) |
| Diabetes mellitus | 57 | 141 | 34.5 | 87.8 | 1.65 | 1.61 | 1.03 (0.75-1.41) |
| Renal disease | 8 | 30 | 5.9 | 16.9 | 1.36 | 1.77 | 0.76 (0.34-1.70) |
| Cancer | 25 | 35 | 9.9 | 26.8 | 2.53 | 1.30 | 1.94 (1.16-3.25) |
| HIV infection | 1 | 0 | 0.2 | - | 4.40 | - | - |

Abbreviations: COVID-19, coronavirus infectious disease 2019; IR, incidence rate; IRR, incidence rate ratio; CI, confidence interval; CCI, Charlson comorbidity index; HIV, human immunodeficiency virus.

**Supplementary Table 3.** Sensitivity analysis on the risk of encephalitis after COVID-19 vaccination by varying the risk window lengths

|  | **No. Event** | | **Person-Years** | | **IR** | | **IRR (95% CI)** |
| --- | --- | --- | --- | --- | --- | --- | --- |
|  | **Risk** | **Control** | **Risk** | **Control** | **Risk** | **Control** |  |
| **Risk window 1-14 days** |  |  |  |  |  |  |  |
| **Overall** | 131 | 665 | 70.2 | 435.8 | 1.87 | 1.53 | 1.22 (1.01-1.46) |
| **Scheduled dose** |  |  |  |  |  |  |  |
| 1^st^ dose | 55 | 665 | 32.3 | 435.8 | 1.71 | 1.53 | 1.12 (0.85-1.47) |
| 2^nd^ dose | 46 | 505 | 26.8 | 293.8 | 1.72 | 1.72 | 1.00 (0.74-1.35) |
| 3^rd^ dose | 30 | 111 | 11.1 | 38.4 | 2.70 | 2.89 | 0.93 (0.62-1.39) |
| **Risk window 1-42 days** |  |  |  |  |  |  |  |
| **Overall** | 352 | 444 | 191.6 | 314.3 | 1.84 | 1.41 | 1.30 (1.13-1.50) |
| **Scheduled dose** |  |  |  |  |  |  |  |
| 1st dose | 142 | 444 | 81.8 | 314.3 | 1.74 | 1.41 | 1.23 (1.02-1.48) |
| 2nd dose | 125 | 373 | 79.8 | 222.6 | 1.57 | 1.68 | 0.94 (0.77-1.14) |
| 3rd dose | 85 | 56 | 30.0 | 19.5 | 2.83 | 2.88 | 0.98 (0.72-1.34) |

Abbreviations: COVID-19, coronavirus infectious disease 2019; IR, incidence rate; IRR, incidence rate ratio; CI, confidence interval.

**Supplementary Table 4.** Sensitivity analysis on the risk of encephalitis after COVID-19 vaccination by varying the case definition

|  | **No. Event** | | **Person-Years** | | **IR** | | **IRR (95% CI)** |
| --- | --- | --- | --- | --- | --- | --- | --- |
|  | **Risk** | **Control** | **Risk** | **Control** | **Risk** | **Control** |  |
| **Restricting to encephalitis cases with a diagnosis record at primary position** | | | | | | | |
| **Overall** | 162 | 317 | 79.4 | 223.2 | 2.04 | 1.42 | 1.44 (1.18-1.75) |
| **Scheduled dose** |  |  |  |  |  |  |  |
| 1^st^ dose | 69 | 317 | 35.9 | 223.2 | 1.92 | 1.42 | 1.35 (1.04-1.76) |
| 2^nd^ dose | 53 | 258 | 30.9 | 150.4 | 1.72 | 1.72 | 1.00 (0.74-1.34) |
| 3^rd^ dose | 40 | 57 | 12.6 | 17.2 | 3.17 | 3.31 | 0.96 (0.66-1.40) |
| **Restricting to encephalitis cases with a prescription record for antivirals, IVIG or systemic corticosteroids** | | | | | | | |
| **Overall** | 211 | 469 | 115.1 | 315.6 | 1.83 | 1.49 | 1.23 (1.05-1.46) |
| **Scheduled dose** |  |  |  |  |  |  |  |
| 1^st^ dose | 88 | 469 | 51.8 | 315.6 | 1.70 | 1.49 | 1.14 (0.91-1.44) |
| 2^nd^ dose | 71 | 379 | 45.3 | 217.8 | 1.57 | 1.74 | 0.90 (0.70-1.16) |
| 3^rd^ dose | 52 | 79 | 18.0 | 24.2 | 2.89 | 3.27 | 0.88 (0.63-1.23) |
| **Including encephalitis cases with a diagnosis made either in-hospital or emergency department settings** | | | | | | | |
| **Overall** | 251 | 545 | 134.8 | 369.3 | 1.86 | 1.48 | 1.26 (1.08-1.47) |
| **Scheduled dose** |  |  |  |  |  |  |  |
| 1st dose | 106 | 545 | 60.2 | 369.3 | 1.76 | 1.48 | 1.19 (0.97-1.47) |
| 2nd dose | 87 | 438 | 53.2 | 256.5 | 1.63 | 1.71 | 0.96 (0.76-1.20) |
| 3rd dose | 58 | 83 | 21.3 | 28.2 | 2.72 | 2.94 | 0.92 (0.67-1.28) |
| **Excluding encephalitis cases identified using diagnosis code for bacterial encephalitis (ICD-10: G04.2)** | | | | | | | |
| **Overall** | 242 | 524 | 123.5 | 345.3 | 1.96 | 1.52 | 1.29 (1.11-1.51) |
| **Scheduled dose** |  |  |  |  |  |  |  |
| 1^st^ dose | 103 | 524 | 55.1 | 345.3 | 1.87 | 1.52 | 1.23 (1.00-1.52) |
| 2^nd^ dose | 83 | 420 | 48.4 | 238.2 | 1.72 | 1.76 | 0.97 (0.77-1.23) |
| 3^rd^ dose | 56 | 82 | 20.0 | 26.8 | 2.80 | 3.06 | 0.92 (0.66-1.27) |

Abbreviations: COVID-19, coronavirus infectious disease 2019; IR, incidence rate; IRR, incidence rate ratio; CI, confidence interval; IVIG, intravenous immunoglobulin; ICD-10, International Classification of Diseases.

**Supplementary Table 5.** Sensitivity analysis on the risk of encephalitis after COVID-19 vaccination in the modified study population

|  | **No. Event** | | **Person-Years** | | **IR** | | **IRR (95% CI)** |
| --- | --- | --- | --- | --- | --- | --- | --- |
|  | **Risk** | **Control** | **Risk** | **Control** | **Risk** | **Control** |  |
| **Excluding encephalitis cases with positive COVID-19 test 90 days prior to COVID-19 vaccination** | | | | | | | |
| **Overall** | 251 | 545 | 134.8 | 369.3 | 1.86 | 1.48 | 1.26 (1.08-1.47) |
| **Scheduled dose** |  |  |  |  |  |  |  |
| 1^st^ dose | 106 | 545 | 60.2 | 369.3 | 1.76 | 1.48 | 1.19 (0.97-1.47) |
| 2^nd^ dose | 87 | 438 | 53.2 | 256.5 | 1.63 | 1.71 | 0.96 (0.76-1.20) |
| 3^rd^ dose | 58 | 83 | 21.3 | 28.2 | 2.72 | 2.94 | 0.92 (0.67-1.28) |
| **Excluding encephalitis cases with positive COVID-19 test prior to encephalitis diagnosis** | | | | | | | |
| **Overall** | 251 | 545 | 128.1 | 357.7 | 1.96 | 1.52 | 1.29 (1.10-1.50) |
| **Scheduled dose** |  |  |  |  |  |  |  |
| 1^st^ dose | 106 | 545 | 57.3 | 357.7 | 1.85 | 1.52 | 1.21 (0.99-1.50) |
| 2^nd^ dose | 87 | 438 | 50.2 | 246.4 | 1.73 | 1.78 | 0.97 (0.78-1.23) |
| 3^rd^ dose | 58 | 83 | 20.6 | 27.7 | 2.82 | 3.00 | 0.94 (0.68-1.30) |
| **Excluding encephalitis cases who died during the 240-day observation period** | | | | | | | |
| **Overall** | 251 | 535 | 133.2 | 365.9 | 1.88 | 1.46 | 1.29 (1.11-1.50) |
| **Scheduled dose** |  |  |  |  |  |  |  |
| 1st dose | 106 | 535 | 59.5 | 365.9 | 1.78 | 1.46 | 1.22 (0.99-1.50) |
| 2nd dose | 87 | 428 | 52.5 | 253.6 | 1.66 | 1.69 | 0.98 (0.78-1.24) |
| 3rd dose | 58 | 82 | 21.2 | 28.2 | 2.73 | 2.91 | 0.94 (0.68-1.30) |

Abbreviations: COVID-19, coronavirus infectious disease 2019; IR, incidence rate; IRR, incidence rate ratio; CI, confidence interval.

**Supplementary Table 6.** Subgroup analysis on the risk of meningitis after COVID-19 vaccination according to the selected characteristics

|  | **No. Event** | | **Person-Years** | | **IR** | | **IRR (95% CI)** |
| --- | --- | --- | --- | --- | --- | --- | --- |
|  | **Risk** | **Control** | **Risk** | **Control** | **Risk** | **Control** |  |
| **Meningitis** |  |  |  |  |  |  |  |
| **Age group** |  |  |  |  |  |  |  |
| 18-29 | 109 | 270 | 75.0 | 172.0 | 1.45 | 1.57 | 0.93 (0.74-1.16) |
| 30-39 | 75 | 181 | 45.2 | 123.6 | 1.66 | 1.46 | 1.13 (0.86-1.49) |
| 40-49 | 57 | 139 | 38.4 | 92.3 | 1.48 | 1.51 | 0.99 (0.72-1.35) |
| 50-59 | 45 | 128 | 31.3 | 79.6 | 1.44 | 1.61 | 0.89 (0.63-1.27) |
| 60-69 | 64 | 122 | 33.7 | 84.3 | 1.90 | 1.45 | 1.31 (0.97-1.78) |
| 70-79 | 34 | 87 | 21.3 | 55.0 | 1.60 | 1.58 | 1.01 (0.67-1.52) |
| 80+ | 14 | 37 | 8.0 | 22.1 | 1.75 | 1.67 | 1.05 (0.58-1.89) |
| **Sex** |  |  |  |  |  |  |  |
| Male | 200 | 503 | 131.1 | 323.0 | 1.53 | 1.56 | 0.98 (0.83-1.16) |
| Female | 198 | 461 | 121.8 | 305.9 | 1.63 | 1.51 | 1.08 (0.91-1.28) |
| **Comorbidities** |  |  |  |  |  |  |  |
| CCI <5 | 357 | 899 | 233.8 | 582.4 | 1.53 | 1.54 | 0.99 (0.87-1.12) |
| CCI ≥5 | 41 | 65 | 19.0 | 46.54 | 2.16 | 1.40 | 1.54 (1.04-2.29) |
| Myocardial infarction | 4 | 7 | 1.7 | 4.70 | 2.31 | 1.49 | 1.55 (0.41-5.80) |
| Congestive heart failure | 20 | 22 | 7.7 | 18.4 | 2.61 | 1.20 | 2.18 (1.20-3.95) |
| Peripheral vascular disease | 31 | 79 | 19.9 | 51.2 | 1.56 | 1.54 | 1.01 (0.66-1.54) |
| Cerebrovascular disease | 26 | 58 | 14.1 | 38.8 | 1.84 | 1.50 | 1.23 (0.77-1.97) |
| Dementia | 19 | 41 | 9.7 | 27.1 | 1.97 | 1.51 | 1.30 (0.75-2.26) |
| Chronic pulmonary disease | 64 | 150 | 39.3 | 98.2 | 1.63 | 1.53 | 1.07 (0.79-1.44) |
| Rheumatic disease | 18 | 26 | 8.8 | 20.8 | 2.04 | 1.25 | 1.63 (0.87-3.03) |
| Peptic ulcer disease | 70 | 138 | 37.7 | 92.1 | 1.86 | 1.50 | 1.24 (0.92-1.66) |
| Hepatic disease | 73 | 188 | 48.0 | 117.4 | 1.52 | 1.60 | 0.95 (0.72-1.25) |
| Diabetes mellitus | 59 | 146 | 38.3 | 92.5 | 1.54 | 1.58 | 0.98 (0.72-1.33) |
| Renal disease | 9 | 27 | 6.3 | 15.0 | 1.43 | 1.80 | 0.80 (0.37-1.71) |
| Cancer | 26 | 43 | 12.8 | 30.6 | 2.03 | 1.41 | 1.44 (0.87-2.38) |
| HIV infection | 1 | 3 | 0.7 | 1.8 | 1.50 | 1.71 | 0.87 (0.11-6.73) |

Abbreviations: COVID-19, coronavirus infectious disease 2019; IR, incidence rate; IRR, incidence rate ratio; CI, confidence interval; CCI, Charlson comorbidity index; HIV, human immunodeficiency virus.

**Supplementary Table 7.** Sensitivity analysis on the risk of meningitis after COVID-19 vaccination by varying the risk window lengths

|  | **No. Event** | | **Person-Years** | | **IR** | | **IRR (95% CI)** |
| --- | --- | --- | --- | --- | --- | --- | --- |
|  | **Risk** | **Control** | **Risk** | **Control** | **Risk** | **Control** |  |
| **Risk window 1-14 days** |  |  |  |  |  |  |  |
| **Overall** | 182 | 1180 | 130.0 | 752.3 | 1.40 | 1.57 | 0.89 (0.76-1.04) |
| **Scheduled dose** |  |  |  |  |  |  |  |
| 1^st^ dose | 81 | 1180 | 55.5 | 752.3 | 1.46 | 1.57 | 0.93 (0.74-1.17) |
| 2^nd^ dose | 67 | 902 | 49.2 | 549.6 | 1.36 | 1.64 | 0.83 (0.65-1.06) |
| 3^rd^ dose | 34 | 173 | 25.4 | 92.4 | 1.34 | 1.87 | 0.72 (0.50-1.03) |
| **Risk window 1-42 days** |  |  |  |  |  |  |  |
| **Overall** | 568 | 794 | 357.0 | 525.3 | 1.59 | 1.51 | 1.05 (0.94-1.17) |
| **Scheduled dose** |  |  |  |  |  |  |  |
| 1st dose | 247 | 794 | 139.3 | 525.3 | 1.77 | 1.51 | 1.17 (1.02-1.35) |
| 2nd dose | 212 | 685 | 147.3 | 409.6 | 1.44 | 1.67 | 0.86 (0.74-1.00) |
| 3rd dose | 109 | 98 | 70.4 | 47.3 | 1.55 | 2.07 | 0.75 (0.57-0.97) |

Abbreviations: COVID-19, coronavirus infectious disease 2019; IR, incidence rate; IRR, incidence rate ratio; CI, confidence interval.

**Supplementary Table 8.** Sensitivity analysis on the risk of meningitis after COVID-19 vaccination by varying the case definition

|  | **No. Event** | | **Person-Years** | | **IR** | | **IRR (95% CI)** |
| --- | --- | --- | --- | --- | --- | --- | --- |
|  | **Risk** | **Control** | **Risk** | **Control** | **Risk** | **Control** |  |
| **Restricting to meningitis cases with a diagnosis record at primary position** | | | | | | | |
| **Overall** | 243 | 611 | 158.3 | 394.1 | 1.54 | 1.55 | 0.99 (0.85-1.15) |
| **Scheduled dose** |  |  |  |  |  |  |  |
| 1^st^ dose | 113 | 611 | 66.3 | 394.1 | 1.70 | 1.55 | 1.10 (0.90-1.34) |
| 2^nd^ dose | 89 | 496 | 60.9 | 296.4 | 1.46 | 1.67 | 0.87 (0.70-1.10) |
| 3^rd^ dose | 41 | 79 | 31.1 | 44.7 | 1.32 | 1.77 | 0.75 (0.52-1.08) |
| **Including meningitis cases with a diagnosis made either in-hospital or emergency department settings** | | | | | | | |
| **Overall** | 398 | 964 | 252.8 | 628.9 | 1.57 | 1.53 | 1.03 (0.91-1.16) |
| **Scheduled dose** |  |  |  |  |  |  |  |
| 1st dose | 178 | 964 | 105.4 | 628.9 | 1.69 | 1.53 | 1.10 (0.94-1.29) |
| 2nd dose | 147 | 784 | 98.3 | 478.1 | 1.50 | 1.64 | 0.91 (0.76-1.09) |
| 3rd dose | 73 | 134 | 49.2 | 68.6 | 1.49 | 1.95 | 0.76 (0.57-1.00) |
| **Excluding meningitis cases identified using diagnosis codes for viral and bacterial meningitis (ICD-10: A87, G04.2)** | | | | | | | |
| **Overall** | 319 | 740 | 186.2 | 474.5 | 1.71 | 1.56 | 1.10 (0.96-1.26) |
| **Scheduled dose** |  |  |  |  |  |  |  |
| 1^st^ dose | 142 | 740 | 77.2 | 474.5 | 1.84 | 1.56 | 1.18 (0.99-1.41) |
| 2^nd^ dose | 117 | 598 | 71.5 | 355.1 | 1.64 | 1.68 | 0.97 (0.80-1.19) |
| 3^rd^ dose | 60 | 103 | 37.5 | 51.6 | 1.60 | 1.99 | 0.80 (0.59-1.10) |

Abbreviations: COVID-19, coronavirus infectious disease 2019; IR, incidence rate; IRR, incidence rate ratio; CI, confidence interval; ICD-10, International Classification of Diseases.

**Supplementary Table 9.** Sensitivity analysis on the risk of meningitis after COVID-19 vaccination in the modified study population

|  | **No. Event** | | **Person-Years** | | **IR** | | **IRR (95% CI)** |
| --- | --- | --- | --- | --- | --- | --- | --- |
|  | **Risk** | **Control** | **Risk** | **Control** | **Risk** | **Control** |  |
| **Excluding meningitis cases with positive COVID-19 test 90 days prior to COVID-19 vaccination** | | | | | | | |
| **Overall** | 398 | 963 | 252.7 | 628.4 | 1.58 | 1.53 | 1.03 (0.91-1.16) |
| **Scheduled dose** |  |  |  |  |  |  |  |
| 1^st^ dose | 178 | 963 | 105.3 | 628.4 | 1.69 | 1.53 | 1.10 (0.94-1.29) |
| 2^nd^ dose | 147 | 783 | 98.2 | 477.5 | 1.50 | 1.64 | 0.91 (0.77-1.09) |
| 3^rd^ dose | 73 | 134 | 49.2 | 68.6 | 1.49 | 1.95 | 0.76 (0.57-1.00) |
| **Excluding meningitis cases with positive COVID-19 test prior to meningitis diagnosis** | | | | | | | |
| **Overall** | 398 | 964 | 239.1 | 608.8 | 1.66 | 1.58 | 1.05 (0.93-1.18) |
| **Scheduled dose** |  |  |  |  |  |  |  |
| 1^st^ dose | 178 | 964 | 99.4 | 608.8 | 1.79 | 1.58 | 1.13 (0.96-1.33) |
| 2^nd^ dose | 147 | 784 | 92.1 | 459.2 | 1.60 | 1.71 | 0.93 (0.78-1.11) |
| 3^rd^ dose | 73 | 134 | 47.6 | 67.2 | 1.53 | 1.99 | 0.77 (0.58-1.02) |
| **Excluding meningitis cases who died during the 240-day observation period** | | | | | | | |
| **Overall** | 398 | 955 | 251.5 | 626.6 | 1.58 | 1.52 | 1.04 (0.92-1.17) |
| **Scheduled dose** |  |  |  |  |  |  |  |
| 1st dose | 178 | 955 | 104.8 | 626.6 | 1.70 | 1.52 | 1.11 (0.95-1.31) |
| 2nd dose | 147 | 776 | 97.7 | 476.1 | 1.51 | 1.63 | 0.92 (0.77-1.10) |
| 3rd dose | 73 | 133 | 49.1 | 68.5 | 1.49 | 1.94 | 0.77 (0.58-1.01) |

Abbreviations: COVID-19, coronavirus infectious disease 2019; IR, incidence rate; IRR, incidence rate ratio; CI, confidence interval.
